# Meat Products: From Animal (Farm) to Meal (Fork)

**DOI:** 10.3390/foods11070933

**Published:** 2022-03-24

**Authors:** Benjamin W. B. Holman, Eric N. Ponnampalam

**Affiliations:** 1 Centre for Red Meat and Sheep Development, NSW Department of Primary Industries, Cowra, NSW 2794, Australia; 2 Animal Production Sciences, Agriculture Victoria Research, Department of Jobs, Precincts and Regions, Bundoora, VIC 3083, Australia

Meat composition and quality are not independent of the effects of animal production systems. The interactions of an animal’s diet (type, form, energy content, nutritional value, etc.), an animal’s physiological status or age, and an animal’s environment will impact the yield, composition, quality, and sensorial appeal of its meat products. If we consider the adoption of forages, novel feeds, industry by-products and more sustainable production systems for holistic benefit, we must also consider their implications for the value of the meat product delivered for human consumption. Consequently, it is important to understand farm (animal/farm) factors that contribute to the quality, expense, and nutritional value of meat. This imperative is a response to the consumers of meat being increasingly aware of the cost, convenience, production system ethics or efficiencies, environmental impacts, food safety and the health or wellness benefits derived from a meat product.

The articles included in this Special Issue have been curated to present findings from different production systems and animal husbandry strategies, in terms of their capacity to deliver meat to consumers that matches their demands.

We begin with an overview of omega-3 and omega-6 fatty acids in red meat, with special reference made to their biochemistry, enhancement by agricultural practice, preservation until the point of consumption, and their recommended dosages within a human diet [1]. The fatty acid and nutraceutical properties of the lipids in the meat of fallow deer, produced under organic and conventional farming systems, were compared [2]. The concentrations of odd- and branched-chain fatty acids in the meat of lamb was compared between different feeding regimes (starch- versus fibre-rich), in an effort to authenticate the production system *post hoc* [3]. Different aroma compounds were identified in the roasted cuts of pre- and post-rigor mutton to understand the preference of some consumers for sheep meat, sold immediately, without chilled storage [4]. The effect of scopoletin supplementation on the quality and antioxidant status of meat from broilers, when held under stocking density stress, was investigated [5]. Breed, gender, season, and geographic region were explored as factors influencing the carcass weight and animal age at slaughter for Greek cattle [6]. The meat quality of Holstein (dairy) cattle was quantified to meet the rising demand for beef in Israel, sustainable production systems, and help address product biases [7]. The maternal prepartum dietary carbohydrate source, during mid- and late- gestation, was explored in terms of its effect on the growth, carcass, and meat quality of the offspring [8]. The supplementation of cattle with distiller’s grains was explored as a means to balance the oxidative stability of beef when displayed under commercial conditions [9]. Finally, the effect of packaging type on the shelf-life, quality and oxidative status of the meat from yearling sheep and young lambs was compared against the effect of the finishing diets (standard diet; standard diet supplemented with camelina forage; or standard diet supplemented with camelina meal) [10].

From these articles, we gain an increased understanding of whole-system approaches and efficacies when managing the transition between animal and meal (i.e., from farm to fork). We are reminded that meat is the result of animal production systems and, more broadly, the agricultural sector. Furthermore, we are focused towards a future where meat production, quality and value are considered within the context of sustainable feed and supplementation selection, the management of animal genetic and husbandry practices, delivery upon market demands and trends, as well as the sustainable processing and packaging of meat products.
